# Research Progress on Tumor-Associated Macrophages and Inflammation in Cervical Cancer

**DOI:** 10.1155/2020/6842963

**Published:** 2020-01-29

**Authors:** Yi Liu, Li Li, Ying Li, Xia Zhao

**Affiliations:** ^1^Department of Gynecology and Obstetrics, Key Laboratory of Birth Defects and Related Diseases of Women and Children, Ministry of Education, West China Second Hospital, Sichuan University, Chengdu 610041, China; ^2^Zunyi Medical University Affiliated Hospital of Obstetrics and Gynecology, Zunyi 563003, China

## Abstract

Cervical cancer is the most common gynecological tumor worldwide. Persistent infection of high-risk HPV-induced smouldering inflammation is considered to be an important risk factor for cervical cancer. The tumor microenvironment (TME) plays an important role in the progress of the tumor occurrence, development, and prognosis of cervical cancer. Macrophages are the main contributor to the TME, which is called tumor-associated macrophages (TAMs). During the inflammatory response, the phenotype and function of TAMs are constantly changing, which are involved in different regulatory networks. The phenotype of TAMs is related to the metabolism and secretory factors release, which facilitate the angiogenesis and lymphatic duct formation during cervical cancer metastasis, thus affecting the prognosis of cervical cancer. This review intends to discuss the recent research progress on the relationship between TAMs and cervical cancer, which is helpful to elucidate the mechanism of TAMs in cervical cancer.

## 1. Introduction

Cervical cancer (CC) is the second most common malignant disease among women in the world, the incidence of CC is increasing year by year, and the age of onset tends to be younger [1]. Current studies have shown that persistent infection of high-risk HPV-16 and HPV-18 is an important risk factor for CC, accounting for 80 to 90% of all cases [2]. The persistent infection of HPV and cervical epithelial dysplasia or cervical intraepithelial neoplasia (CIN) exist at the same time. If the diagnosis and treatment were not standardized in time, there will be different degrees of CIN developing into invasive cancer. Studies have shown that high-risk HPV infection, such as HPV16/18, was prone to the integration of the HPV gene into the genome, interfering with the immune response of the body and the accumulation of microlesions, which finally developed into CC [3]. Recent studies have shown that smouldering inflammatory response and oxidative stress caused by persistent HPV infection play an important role in the process leading to cervical cancer, and HPV-induced epigenetic changes also play an important role [4, 5]. HPV virus infection can lead to inflammation in the human body, and the occurrence of cervical cancer is also considered to be a process of smouldering inflammation [6].

Inflammatory cells act as a bridge between tumor and inflammation. There are a large number of inflammatory cells around the tumor cells, including macrophages, dendritic cells, and mast cells. These inflammatory cells form tumor microenvironment (TME) with tumor cells and vascular endothelial cells [7]. The “seed and soil” hypothesis put forward by Stephen Paget in 1889 is the basis of the concept of the tumor microenvironment, which accurately predicts that tumor cells as “seeds” can settle in the “soil” suitable for their growth, that is, distal tissues and organs [9]. Tumor cells must work in synergy with their surroundings. Macrophages that play the most important role in the tumor microenvironment were called tumor-associated macrophages (TAMs), accounting for 30% to 50% of the TME cells. TAMs in the tumor microenvironment are closely related to tumor development and participate in biological processes such as angiogenesis, tumor cell invasion, migration and intravascular perfusion, and inhibition of antitumor immune response, to promote the progress to the malignant tumor [10, 11]. The study on the relationship between TAMs in cervical cancer is the focus of research in recent years. The change of microenvironment leads to alternative polarization of macrophages and the change of biological function, which plays an important role in the development of cervical cancer.

This review systematically collects the research on the relationship between tumor-related macrophages in cervical cancer published in recent years, in order to understand the relationship between tumor-related macrophages and the occurrence and development of cervical cancer and in order to contribute to the more effective prevention and treatment of cervical cancer.

## 2. Source of Tumor-Associated Macrophages

Researches show that the TME refers to the environment around a tumor cell [12]. A large number of factors are produced around tumor cells, such as CSF-1, granulocyte macrophage colony-stimulating factor, transforming growth factor (TGF)-*β*-1, and macrophage stimulating protein (MSP), as well as various enzymes. The integrated system of these cells, factors, and enzymes constitutes the microenvironment of tumor tissue [13]. Compared with the normal tissue microenvironment, the tumor tissue microenvironment has the characteristics of low oxygen content, low pH, and higher cell interstitial pressure [14].

Macrophages are the main cells of the innate immune system. Macrophages are widely found in many tissues of the human body, such as bone marrow, connective tissue, alveolar macrophages in the lung, Kupffer cells in the liver, spleen and lymph nodes, serous cavity (abdominal cavity, pleural cavity, and pericardial cavity), bone tissue (osteoclast), nerve tissue (microglia), and skin (Langerhans cell) [15]. Macrophages are important representatives of immune function in the progress of cancer, an important part of host defense, and an antigen-presenting cells and effector cells [16]. Most TAMs come from peripheral blood monocytes from bone marrow and differentiate into different macrophage subsets in TME [17]. TAM is an important inflammatory response cell in the tumor tissue microenvironment, which has high plasticity and plays an important role in the regulation of immune function in tumor tissue [18]. Macrophages in normal or inflammatory tissues exhibit spontaneous antitumor activity, while TAMs can be used to promote tumor growth, reshape tissue, promote angiogenesis, and inhibit acquired immunity.

## 3. TAM Polarization

Macrophages are inherent immune cells in humoral immunity in the human immune system and play an important role in the autoimmune and inflammatory response and tumor immunity [19]. The bidirectional effect of lymphocytes and other inherent lymphoid cell subsets occurred during the resolution of inflammation and wound healing of macrophages after infection or injury [20, 21]. Cancer cells recruit TAMs into inflammatory TME [22], and recruited TAMs increase migration and maintain the dryness of cancer cells [23]. According to the different phenotypes of TAM, it can be divided into M1 and M2 macrophages [10]. The activation pathways are the classical activation pathway and alternative activation pathway, respectively. There is a continuum between classical and alternative activation of macrophages. The classical activation pathway was defined as that cytokine interferon *γ* (INF-*γ*) or bacterial products (such as lipopolysaccharide (LPS)) stimulate the differentiation of Th1 into macrophages. Macrophages caused by the action of Th2-related cytokines, IL-4, IL-13, IL-10, TGF-*β*, and anti-inflammatory factors such as glucocorticoids and vitamin A belong to alternative activation pathways [24]. Polarized macrophages to M1 phenotypic macrophages are characterized by their ability to release proinflammatory cytokines such as tumor necrosis factor-*α* (TNF-*α*), IL-1, IL-6, IL-12, IL-23, active nitrogen, and oxygen intermediates. The high expression of major histocompatibility complex II has antigen presentation and bactericidal and tumor-killing activity, which hinders the progress of the tumor. M2 is a substitute for activated macrophages, secreting cytokines such as IL-4, IL-13, IL-10, vitamin D3, and glucocorticoid. Low expression of IL-12 and high expression of IL-10, transforming growth factor-*β* (TGF-*β*), angiogenic factor, scavenger and mannose, galactose receptor, CD163, and CD206 were characterized by high expression [25, 26]. CD206, along with Arg-1 and Ym1, is known as the classic marker of M2 cells [27]. The M2 antigen presentation ability is weakened, the antitumor activity is low, and the ability to support angiogenesis and tissue remodeling is enhanced, which is beneficial to tumor growth and invasion [28, 29]. High levels of FGF-2, MCP-1, CXCL12, and VEGF in TME were associated with an increase in the number of M2 cells [26, 30]. It will be a new research direction in the future to develop a new therapy for tumors by targeting TAMs [31].

## 4. The Role of TAMS in Carcinogenesis

In the middle of the 19th century, German scientist Rudolf Virchow proposed the hypothesis that smouldering inflammation can lead to cancer [32]. The main function of macrophages is the regulation of inflammation; with the deepening of research, the relationship between inflammation, innate immunity, and tumorigenesis has been widely recognized [33, 34], but there are still many mechanisms that are not fully understood. In the process of tumorigenesis, TAMs in the microenvironment are beneficial to the survival and proliferation of cancer cells. Macrophages stimulate inflammation and then promote tumorigenesis. It has been reported that more than 15% of malignant tumors may be indirectly related to specific infections [35–37], such as the occurrence of gastric cancer, esophageal cancer, and cervical cancer. Smouldering inflammation caused by persistent infection is regulated by a variety of regulatory mechanisms. Inflammatory cells form a new microenvironment by secreting various cytokines such as inflammatory factors, chemokines, adhesion molecules, and extracellular matrix. Tumor microenvironment composed of inflammatory cells, inflammatory factors and their mediators, chemokines, and extracellular matrix plays an important role in the proliferation, survival, and metastasis of tumor cells [38]. Inflammatory molecules involved in inflammation-mediated cervical cancer include reactive oxygen species (ROS), TNF-*α*, IL-1, IL-6, IL-8, IL-18, hypoxia-inducible factor (HIF), cyclooxygenase (COX), inducible nitric oxide synthase (INOS), Matrix metalloproteinase enzyme-9 (MMP-9), and chemokines [39].

Unlike physiological dampening inflammation, smouldering inflammation is caused by the persistence of factors that initiate inflammation. The persistent inflammatory response leads to the presence of highly active cytokines and compounds, leading to the formation of peroxynitrite, a mutagen. In smouldering inflammation, there are two processes in tissues: tissue damage caused by inflammation-activated pathogens and/or bactericidal activity of macrophages and stimulation of regeneration. This leads to increased proliferation of epithelial cells in the context of high concentrations of mutagenic compounds, increasing the probability of mutation, leading to such genomic aberrations as point mutations, deletions, and rearrangements. Extracellular matrix components stimulate angiogenesis [40]. Feedforward circulation increases the sustainability of inflammation; tumor cells and tumor-related macrophages regulate inflammation by secreting matrix metalloproteinases, cytokines, chemokines, growth, and angiogenesis factors [33]. In the case of inflammatory bowel diseases such as ulcerative colitis and Crohn's disease, the strongest correlation between smouldering inflammation and the development of malignant tumors was observed. Smouldering hepatitis C virus infection also increases the risk of hepatocellular carcinoma. Smouldering inflammation caused by *Helicobacter pylori* increases the risk of gastric cancer [41]. TAMs have high plasticity, which adapts to environmental changes through changes in cell metabolism and immunophenotype. TAMs have different phenotypes in different anatomical locations and physiological characteristics. In the early stage of tumorigenesis, macrophages participate in tumorigenesis by producing activated free radicals, which lead to DNA damage of tumour cells [42].

In different stages of cervical cancer, the phenotype of macrophages is constantly changing [43], which affects the ability of proliferation, invasion, and metastasis of cancer tissue in many ways [44]. The essence of TAM transformation between two different phenotypes is the transformation between different immune types of tissue cells in different stages of cancer development. The DNA damage is the main mechanism leading to malignant tumors in the context of smouldering inflammation. However, the mechanism of DNA damage is due to the secretion of cytokines and the interaction between inflammatory infiltrating cells. Macrophage inhibitory factor (MIF) produced by macrophages inhibits P53 activity [45], activates the antiapoptotic pathway of cells, leads to the increase of cell life, and increases the probability of DNA damage. It leads to the increase and accumulation of more meaningful mutations.

Hypoxia is a characteristic of the tumor microenvironment. Hypoxia-inducible factors usually exist in tissues. Oxygen levels are maintained by steady-state mechanisms at cellular, organ, and system levels. The percentage of oxygen in normal tissues ranges from 22.5% to 9%. However, the fast-growing tumor will form hypoxic regions due to low blood supply (poor vascularization or disorganized vessel structure), and a large number of infiltrating cells will reduce the oxygen levels to less than 1%. During the inflammatory response, hypoxia-inducible factor 1*α* (HIF-1*α*) is activated and then activates the production of vascular endothelial growth factor (VEGF). The stimulation of macrophage M-CSF enhanced the production of VEGF [46]. In addition to the angiogenesis activity, VEGF has the chemical attractant properties of macrophages [47, 48], thus forming positive feedback to accelerate angiogenesis of tumors. Macrophages are the main source of TNF-*α* and IL-1, and their expression increases with the invasion of tumors [49, 50] and angiogenesis [51, 52]. HIF triggers other inflammatory mediators and proteases to participate in tumorigenesis and plays a major role in cell transformation, promotion, survival, proliferation, invasion, angiogenesis, and metastasis [53, 54]. In cervical cancer cells, it induces amphiphilic regulatory proteins, which in turn play a major role in proliferation [55, 56]. IL-1, IL-6, IL-8, and IL-18 are involved in the inflammatory process [57], IL-1 and IL-6 are involved in cell growth, metastasis, and tumor development [58].

## 5. Changes of TAMs during the Development of Cervical Cancer

It has been found that TAMs are closely related to the development of cervical intraepithelial neoplasia in the course of cervical cancer. In the process of developing from chronic cervicitis to CIN I-III and finally to invasive CC, the expression of macrophages and new blood vessels in tumor microenvironment increases synchronously. In the development and progress of cancer cells, TAMs and tumor angiogenesis are important and closely related [59]. The number of TAMs in cervical lesion matrix changes with the progress of cervical cancer. M1 macrophages release inflammatory factors, upregulate immune response, and inhibit the occurrence of cervical cancer. After the treatment of M1 macrophages with squamous epithelial cells of cervical cancer, it can be seen that the phenotype of macrophages transforms from M1 to M2. In cervical cancer, the phenotype of TAMs is regulated by lactate secreted by cervical cancer cells [60]. Polarization of tumor-associated macrophages toward the M2 phenotype correlates with poor response to chemoradiation and reduces survival in patients with locally advanced cervical cancer [61]. M2 increases the expression of CD163 and IL-10. CD163, as a promising TAMs marker, is superior to CD68 for predicting the malignant transformation and metastatic potential of cervical cancer [62]. IL-10, as an immunosuppressive factor closely related to the occurrence and development of cervical cancer, is mainly regulated during transcription, inhibiting the killing effect of the immune system on tumors in various ways and promoting the occurrence, development, and metastasis of tumors [63–65]. The expression of the IL-10 gene is mainly through the combination of a series of transcription factors in the promoter region. These transcription factors include Ets1, SP1, CCAAT/enhancer-binding protein *β*, and STAT3 [66]. IL-10 downregulates the immune response and promotes tumor progression. The transformation of TAMs in the cervical cancer microenvironment belongs to the transformation of the immune type, which plays a key role in the prognosis of cervical cancer.

## 6. Therapeutic Interventions Targeting TAMs in CC

In recent, more and more attention has been paid to the relationship between TAMs and tumor prognosis. Studies suggested that the increase of TAMs infiltration is associated with the increase in the poor prognosis of patients [67, 68]. A survey of 101 CC patients at stage IB and stage IIA showed that the increase of CD163+ macrophage count was significantly related to the decrease of recurrence-free survival rate [69]. Nedergaard et al. reported that there was no significant relationship between CD68+ macrophages and tumor recurrence in stage I squamous cell carcinoma of cervix uteri [70]. Chen et al. found that the expressions of TAMs CD68+ and CD163+ were positively correlated with the occurrence of CC in patients with high-risk HPV infection and that the correlation between CD163+ macrophage level and lymph node metastasis in patients with advanced CC was stronger than that of CD68+ macrophage [62]. Thus, TAMs could be used as the treatment target in the treatment of cervical cancer, which can inhibit the activation of M2 macrophages, prevent recruitment of monocytes into the tumor, activate the phagocytic activity of macrophages, increase the activation of T cells, and finally improve the prognosis of patients.

## 7. Summary

**Figure 1:**
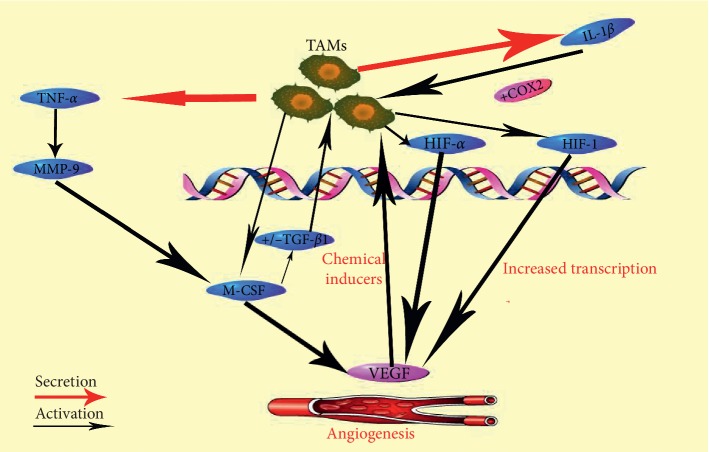
The role of TAMs in cervical cancer.

The role of TAMs in malignant tumors has become increasingly clear with the continuous research in recent years, and its role in tumors has attracted more and more attention. The specific surface molecules of TAMs, as tumor markers, can be used as diagnostic and prognostic markers for various tumors, providing research directions for finding new targeted therapeutic drugs in the future. Studying the various mechanisms (Figure 1) of the impact of tumor microenvironment on tumorigenesis and progression makes it possible to find new targets for specific anticancer therapy and prevention of cancer cell proliferation, which creates prospects for the progress of anticancer.
